# Non-classicality and the effect of one photon

**DOI:** 10.1098/rsta.2023.0331

**Published:** 2024-12-24

**Authors:** Christopher C. Gerry, Richard J. Birrittella, Paul M. Alsing, Jihane Mimih, Peter L. Knight

**Affiliations:** ^1^ Department of Physics and Astronomy, Lehman College, The City University of New York, Bronx, NY 10468-1589, USA; ^2^ Booz Allen Hamilton, McLean, VA 22102, USA; ^3^ University at Albany-SUNY, Albany, NY 12222, USA; ^4^ Department of Electrical and Computer Engineering, Naval Postgraduate School, Monterey, CA 93943, USA; ^5^ Blackett Laboratory, Imperial College, London SW72AZ, UK

**Keywords:** quantum, non-classical, quantum interference

## Abstract

The quantum interference effects of mixing the most non-classical states of light, number states, with the most classical-like of pure field states, the coherent state, are investigated. We demonstrate how the non-classicality of a single photon when mixed with a coherent field can transform the statistical properties of the output and further demonstrate that the entanglement of the output is independent of the coherent state amplitude.

This article is part of the theme issue ‘The quantum theory of light’.

## 1. Introduction

Rodney Loudon was a pioneer of quantum optics with many notable achievements demonstrating the non-classical effects of quantized radiation fields [1,2]. One of his most important contributions was the study of interference between mode amplitudes [3,4] building, in part, on earlier work by Feynman *et al*. in 1965 [5,6], which led to a fruitful experimental investigation led by Rarity and his group [7,8]. At roughly the same time, Mandel and his group were the first to demonstrate the unusual properties of two-photon interference [9] which underpin much of recent quantum optics [10,11].

In this paper, we address the quantum interference effects of mixing the most non-classical states of light, that being number (or Fock) states, with the most classical-like of pure states of the field, the coherent state [12]. In particular, we consider the mixing of coherent states of arbitrary amplitude with a single photon at a balanced 50 : 50 beam splitter. For a coherent state, the average photon number of the state could be a macroscopically large number. Should $\bar{n}$ be in the billions, intuition would probably suggest that mixing in a single photon would have little effect on the output state of the beam splitter. Such intuition is dramatically wrong, as we shall demonstrate below. For a coherent state mixed with a vacuum state at a 50 : 50 beam splitter, the output is just a product of two coherent states for which the joint photon-number distribution is just a product of two Poissonian distributions and consists of a single peak centred around $n_{1}=n_{2}=\bar{n}/2$. But by mixing coherent light with a single photon, the joint photon-number distribution of the output state becomes symmetrically bimodal around a ‘diagonal’ line of zeros following $n_{1}=n_{2}$. This line of zeros, referred to as the central nodal line [13], is present regardless of the strength of the input coherent light beam.

Before we start to present our argument, it is worthwhile to remove some misconceptions. The term ‘multi-photon interference’ appears often in the physics literature [14]. Taken at face value, the term would lead one to believe that photons (perhaps many) are somehow interfering with other photons. On the other hand, according to Dirac in a famous passage on the Young double-slit experiment in the fourth edition of his book *Quantum Mechanics* [15], where he discusses interference with only one photon at a time passing through the apparatus, ‘... each photon interferes only with itself. Interference between different photons never occurs.’ Unfortunately, but unsurprisingly, this statement has led to much confusion, especially in connection with attempts to reconcile it with the results of experiments that showed that light from separate lasers could produce interference effects [16]. Subsequently, the issue was succinctly addressed in a letter by Glauber [17] in 1995, wherein he pointed out that the Dirac statement is misleading and fundamentally incorrect even though it has been elevated to the ‘level of scripture’ [17]. In one sense, the Dirac statement *is* correct in that photons do not interfere with other photons. But it is wrong to say that ‘each photon interferes with itself.’ In reality, a photon does not interfere with other photons *nor* with itself. Photons do not interfere at all. The Dirac statement, as Glauber points out, was mainly directed at the simplistic notion of photons ‘eating or reinforcing one another’ [17]. Rather, it is the quantum probability amplitudes associated with different processes for obtaining a particular outcome that interfere, with the modulus of the sum squared being the corresponding probability, as was discussed by Feynman *et al*. [6]. The phrase ‘multi-photon quantum interference’ should be understood to mean interference by states containing multiple numbers of photons.

**Figure 1.**
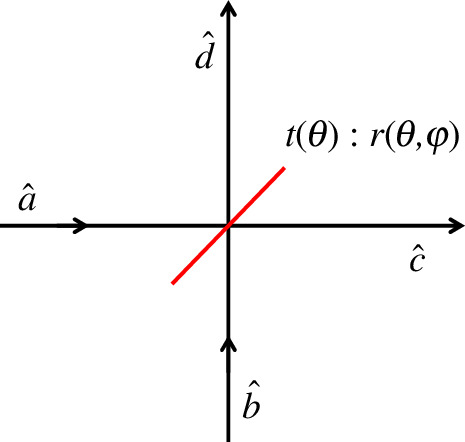
Schematic detailing the mode labelling for a quantum mechanical beam splitter. Note that both the transmittance t and reflectance r are dependent upon the beam splitter angle θ.

The simplest example of a ‘multi-photon quantum interference’ is the Hong–Ou–Mandel (HOM) effect [9], which involves two indistinguishable photons simultaneously falling on opposite sides of a beam splitter as shown in figure 1. Through quantum interference, the photons always emerge together in the same beam, though which beam will contain the photons on a given run of the experiment is completely random. The origin of the effect stems from the fact that there are two possible ways by which one photon could be in each of the output beams: both photons can be transmitted through the beam splitter or both photons can reflect off the beam splitter, with these processes being of equal amplitude but different signs, resulting in destructive interference of the equal photon-number outcome.

The experimental demonstration of this effect [9] involved using type-I non-degenerate parametric down conversion [10], which to first order generates twin single photons in the signal and idler beams, which are then directed to opposite sides of a 50 : 50 beam splitter. By adjusting the relative path lengths to the beam splitter, the coincidence counting rate observed by single-photon detectors placed in the output beams is brought to zero. The dip in the coincidence counting rate is often considered to *be* the HOM effect, but we consider the lack of single photons in each output beam because of quantum interference to be the actual HOM effect, which occurs only at the bottom of the HOM dip. The dip in the coincident rate is the result of what one must do as preparation to *observe* the HOM effect.

## 2. Beam splitter fundamentals and the HOM effect

The quantum mechanics of the beam splitter is described in many books and papers (for a fairly comprehensive list of references, see [10]). The beam splitter transformations, which are required to preserve the commutation relations, are given by


(2.1) $$\hat{c}=t^{\prime }\hat{a}+r\hat{b},\;\;\;\;\;\;\;\;\hat{d}=r^{\prime }\hat{a}+t\hat{b},$$

where the transmissivity and reflectivity are given, respectively, by $t=t^{\prime }=\cos\;\frac{\theta }{2}$ and $r=-r^{\prime \;*}=e^{i\varphi }\;\sin\;\frac{\theta }{2}$ such that $T=|t{|}^{2}=\cos^{2}\;\frac{\theta }{2}$ and $R=|r{|}^{2}=\sin^{2}\;\frac{\theta }{2}$, where T and R are the transmittance and reflectance, respectively, and the angle θ parameterizes the transmittance/reflectance and φ characterizes the phase shift of the reflected beam. A schematic detailing the mode labelling is shown in figure 1. In terms of these angles, the beam splitter transformations are given by


(2.2) $$\begin{array}{ll} \hat{c} & =\hat{a}\;\cos\;\frac{\theta }{2}+\hat{b}e^{i\varphi }\;\sin\;\frac{\theta }{2},\\ \hat{d} & =-\hat{a}e^{-i\varphi }\;\sin\;\frac{\theta }{2}+\hat{b}\;\cos\;\frac{\theta }{2}. \end{array}$$

These transformations can be written in the compact matrix form


(2.3) $$\left(\begin{array}{c} \hat{c}\\ \hat{d} \end{array}\right)={\hat{U}}^{†}(\theta ,\varphi )\left(\begin{array}{c} \hat{a}\\ \hat{b} \end{array}\right)\hat{U}(\theta ,\varphi ),$$

where the unitary operator is given by


(2.4) $$\hat{U}(\theta ,\varphi )=\text{exp}\;\left[\frac{\theta }{2}\left({\hat{a}}^{†}\hat{b}e^{i\varphi }-\hat{a}{\hat{b}}^{†}e^{-i\varphi }\right)\right].$$

By using the Baker-Hausdorff lemma in equation (2.4) one again arrives at equation (2.2). A 50 : 50 beam splitter that imparts a $\frac{\pi }{2}$-phase shift to the reflected beam is characterized by the angles $\theta =\varphi =\frac{\pi }{2}$, such that


(2.5) $$\hat{c}=\frac{1}{\sqrt{2}}\left(\hat{a}+i\hat{b}\right),\;\;\;\hat{d}=\frac{1}{\sqrt{2}}\left(\hat{b}+i\hat{a}\right).$$

Note that the corresponding reflected modes above pick up a $\frac{\pi }{2}$-phase shift. Inverting equation (2.5) it is straightforward to work out


(2.6) $$\begin{array}{ll} {\left|1,1\right\rangle }_{a,b}={\hat{a}}^{†}{\hat{b}}^{†}{\left|0,0\right\rangle }_{a,b} & \to \frac{1}{2}\left({\hat{c}}^{†}+i{\hat{d}}^{†}\right)\left({\hat{d}}^{†}+i{\hat{c}}^{†}\right){\left|0,0\right\rangle }_{c,d}\\ & =\frac{i}{2}\left({\hat{c}}^{†2}+{\hat{d}}^{†2}\right){\left|0,0\right\rangle }_{c,d}\\ & =\frac{i}{\sqrt{2}}\left({\left|2,0\right\rangle }_{c,d}+{\left|0,2\right\rangle }_{c,d}\right), \end{array}$$

where it is clear that the two probability amplitudes associated with obtaining the ${|1,1\rangle }_{c,d}$ output destructively interfere. This quantum interference is the essence of the well-known HOM effect.

## 3. Mixing a single photon with a coherent state

The HOM effect is a microscopic effect, in that it involves states each containing only one photon, as demonstrated in the previous section. The state obtained is an entangled state. On the other hand, mixing light prepared in coherent states (classical-like states of light as emitted by a phase-stabilized laser) at a beam splitter does not result in entanglement; it results in just a product of coherent states. Generating entanglement using a beam splitter requires that at least one of the input states be a non-classical state of light [18]. Non-classical states of light could be discrete states, i.e. number states, or continuous states of light such as the single-mode squeezed vacuum state or squeezed coherent states [19]. Photon-number states are, in a sense, the most non-classical states of the quantized electromagnetic field in that their associated Wigner functions take on negative values over large regions of phase space [10]. The Wigner functions are non-Gaussian. On the other hand, the squeezed vacuum and squeezed coherent states, which can be macroscopically occupied, possess Gaussian Wigner functions. They are positive everywhere in phase space, yet the corresponding states are non-classical owing to the squeezing of the quantum noise in the field quadrature operators [10].

Since one of the states being mixed is highly non-classical and non-Gaussian, the two-mode output state obtained by mixing one photon with coherent light will be non-classical and non-Gaussian as well. It is expected to be an entangled state. Sekatski *et al*. [20] put forward a proposal for exploring ‘macroscopic entanglement’ by mixing a single photon with coherent light of large amplitude. However, as was shown numerically by Birrittella [21] and Birrittella *et al*. [22] the degree of entanglement obtained is independent of the amplitude of the coherent state and, in fact, is exactly the same as that obtained for a single photon mixed with a vacuum state at a 50 : 50 beam splitter. The proof of why this is the case will be demonstrated in this paper. Of course, local operations cannot increase entanglement and the generation of a coherent state by a displacement operator acting on just one of the modes is just such a local operation.

Around the same time as the paper by Birrittella *et al.* [22] and that of Sekatski *et al.* [20], there appeared a paper by Windhager *et al.* [23] which considered one photon mixing with a coherent state, and one by Xu *et al.* [24] which considered an arbitrary photon-number state mixing with a coherent state. Neither of these papers examined the joint photon-number distribution resulting from quantum interference effects.

**Figure 2.**
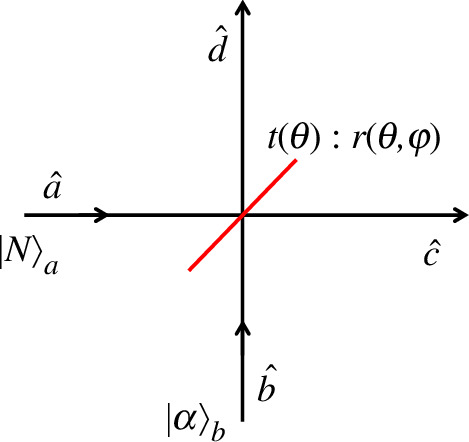
Schematic detailing the mixing of N photons in the a-mode with a coherent state of amplitude α in the b-mode. Note that both the transmittance t and reflectance r are dependent upon the beam splitter angle θ.


As alluded to earlier, an interesting interference effect occurs akin to an ‘extended’ HOM effect wherein destructive interference can be seen in the joint photon-number distribution along the diagonal line $n_{1}=n_{2}$ provided one of the input number state is of odd parity, i.e. |2k+1⟩,k=0,1,2,…. As a simple demonstration, consider the input state ${|N\rangle }_{a}⊗{|\alpha \rangle }_{b}={|N,\alpha \rangle }_{a,b}$ incident upon a beam splitter. A schematic of the system is shown in figure 2. Assuming a 50:50 beam splitter in what follows, the output state is found to be

(3.1) $$\begin{array}{ll} {\left|\text{out}\right\rangle }_{a,b}=\hat{U}{\left|N,\alpha \right\rangle }_{a,b} & =\hat{U}{\hat{D}}_{b}(\alpha ){\left|N,0\right\rangle }_{a,b}=\left[\hat{U}{\hat{D}}_{b}(\alpha ){\hat{U}}^{†}\right]\left(\hat{U}{\left|N,0\right\rangle }_{a,b}\right), \end{array}$$

where $\hat{U}$ is the beam splitter transformation and ${\hat{D}}_{i}$ is the displacement operator acting on the ith mode. But we know that $\hat{U}{\hat{D}}_{b}(\alpha ){\hat{U}}^{†}$ will result in a product of displacement operators:


(3.2) $$\hat{U}\hat{D}(\alpha {)}_{b}{\hat{U}}^{†}={\hat{D}}_{a}(\mu )⊗{\hat{D}}_{b}(\nu ),$$

where the new displacement amplitudes are $\mu =\alpha /\sqrt{2}\;\text{and}\;\nu =i\alpha /\sqrt{2}$, for the *a* and b modes, respectively. Our output state vector is now written as


(3.3) $$\left|\text{out}\right\rangle ={\hat{D}}_{a}(\alpha /\sqrt{2}){\hat{D}}_{b}(i\alpha /\sqrt{2})\left(\hat{U}{\left|N,0\right\rangle }_{a,b}\right).$$

Carrying out the transformation in parentheses above yields [25]


(3.4) U^|N,0⟩a,b=(12)N∑k=0Nik(Nk)1/2|N−k,k⟩a,b.

Plugging this into equation (3.3), resolving unity twice and projecting on to the state ${\left|M,M\right\rangle }_{a,b}$, one obtains the corresponding probability amplitude γ(M,M):


(3.5) γ(M,M)=⟨M,M|out⟩=∑n=0Nin(Nn)1/2⟨M,M|D^a(α/2)D^b(iα/2)|N−n,n⟩.

For the simple case of N=1, and setting $\tilde{\alpha }=\alpha /\sqrt{2}$ for notational convenience, this result simplifies tremendously to


(3.6) $$\begin{array}{ll} \gamma (M,M) & =\frac{1}{\sqrt{2}}\left(\langle M,M|{\hat{D}}_{a}(\tilde{\alpha }){\hat{D}}_{b}(i\tilde{\alpha })|1,0\rangle +i\langle M,M|{\hat{D}}_{a}(\tilde{\alpha }){\hat{D}}_{b}(i\tilde{\alpha })|0,1\rangle \right)\\ & =\frac{1}{\sqrt{2}}\left(\langle M,M|{\hat{D}}_{a}(\tilde{\alpha }){\hat{D}}_{b}(\tilde{\alpha })|1,0\rangle \;i^{M-1}+i\langle M,M|{\hat{D}}_{a}(\tilde{\alpha }){\hat{D}}_{b}(\tilde{\alpha })|0,1\rangle \;i^{M}\right)\\ & =\frac{i}{\sqrt{2}}\langle M|\tilde{\alpha }\rangle \langle M|\hat{D}(\tilde{\alpha })|1\rangle \;\left(i^{-1}+i\right)=0, \end{array}$$

where we dropped the subscripts in the last step as the magnitude of both terms in the sum were the same and consequently factor out, and where we used the identities


(3.7) $$\langle M|i\beta \rangle \;=\;i^{M}\;\langle M|\beta \rangle ,\;\;\;\;\;\;\;\;\;\;\;\langle M|\hat{D}(i\beta )|1\rangle =i^{M-1}\;\langle M|\hat{D}(\beta )|1\rangle .$$

**Figure 3.**
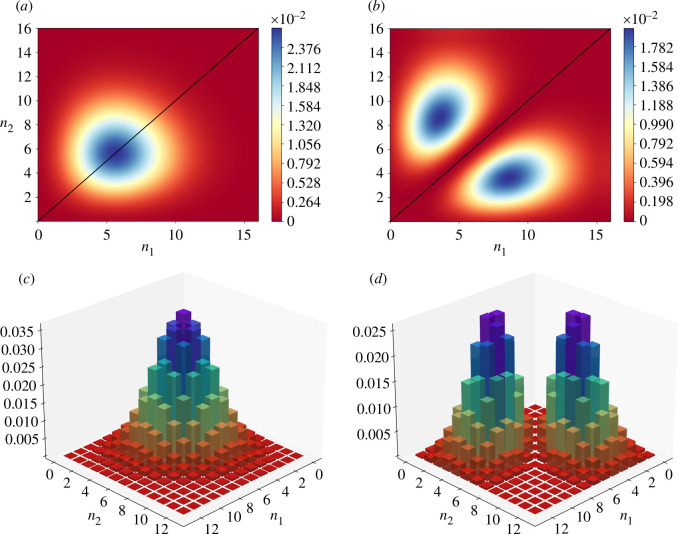
(interpolated) Contours of the joint photon-number distributions for the case of (*a*) coherent light incident at 50 : 50 beam splitter with a vacuum state and (*b*) coherent light mixed with a single photon at a beam splitter. We include the corresponding bar chart probability distributions in (*c*) and (*d*), respectively. The presence of a single photon results in a bimodal distribution with a line of destructive interference along the diagonal (black) line corresponding to $n_{1}=n_{2}$.

The effect of this destructive interference on the joint photon-number distribution is shown in figure 3 where we consider mixing a coherent state with both a vacuum and a single photon, the latter case showcasing the destructive interference of the correlated states. We note that while we carried out the derivation for the input 1-photon state, this will hold true for any odd input Fock state. In reality, the effect is not limited to coherent states mixed with number states. As long as one of the input states to the beam splitter is definite odd parity, all the M-photon correlated states will vanish by destructive interference. This is referred to in the literature as the extended HOM effect (eHOM) [13].

## 4. Proof that the entanglement is independent of the coherent state amplitude

To demonstrate the degree of entanglement let us return to the input state of equation (3.1) but for the case in which a single photon is initially in the a mode:


(4.1) $$\begin{array}{ll} {\left|\text{out}\right\rangle }_{a,b} & =\hat{U}{\left|1,\alpha \right\rangle }_{a,b}=\hat{U}{\hat{D}}_{b}(\alpha ){\left|1,0\right\rangle }_{a,b}=\left[\hat{U}{\hat{D}}_{b}(\alpha ){\hat{U}}^{†}\right]\left(\hat{U}{\left|1,0\right\rangle }_{a,b}\right)\\ & ={\hat{D}}_{a}(\mu )\;{\hat{D}}_{b}\;(\nu )\;[\frac{1}{\sqrt{2}}\left({\left|1,0\right\rangle }_{a,b}+i{\left|0,1\right\rangle }_{a,b}\right)], \end{array}$$

where μ and ν are defined in §3. To address mode entanglement, it is sufficient to calculate the reduced density operator for each mode, and then evaluate the trace of its square as a measure of the linear entropy, i.e. $S_{\text{lin.}}=1-{\text{Tr}}_{b}[\rho_{a}^{2}]=1-P_{a}$, where $P_{a}$ is the a-mode purity, and hence identify how entangled the modes become as a result of mixing at the beam splitter. Defining $\hat{d}={\hat{D}}_{a}\;(\mu )\;{\hat{D}}_{b}\;(\nu )$, the corresponding density operator is (dropping state subscripts)


(4.2) $$\begin{array}{ll} \rho_{\text{out}} & =\frac{1}{2}\left(\hat{d}\left|1,0\right\rangle \;\left\langle 1,0\right|{\hat{d}}^{†}+\hat{d}\left|0,1\right\rangle \;\left\langle 0,1\right|{\hat{d}}^{†}\right)+\frac{i}{2}\left(\hat{d}\left|0,1\right\rangle \;\left\langle 1,0\right|{\hat{d}}^{†}-\hat{d}\left|1,0\right\rangle \;\left\langle 0,1\right|{\hat{d}}^{†}\right). \end{array}$$

The reduced density operator for the a-mode is given by the usual expression


(4.3) $$\rho_{a}={\text{Tr}}_{b}[\rho_{\text{out}}]=\sum_{m=0}^{\infty }{\;}_{b}\langle m|\rho_{\text{out}}|m\rangle_{b}.$$

Using the completeness relation for the number states, $\sum_{n=0}^{\infty }\left|n\right\rangle \left\langle n\right|=\hat{I}$, and the orthonormality of the vacuum and single-photon states, it is straightforward to show that


(4.4) $$\rho_{a}=\frac{1}{2}{\hat{D}}_{a}(\mu )[{\left|0\right\rangle }_{a}\left\langle 0\right|+{\left|1\right\rangle }_{a}\left\langle 1\right|]{\hat{D}}_{a}^{†}(\mu ).$$

This comes from tracing the first term of equation (4.2) in parentheses (the second term in parentheses traces to zero). Next, we need to evaluate the square of the reduced density operator, yielding


(4.5) $$\rho_{a}^{2}=\frac{1}{4}{\hat{D}}_{a}(\mu )[{\left|0\right\rangle }_{a}\left\langle 0\right|+{\left|1\right\rangle }_{a}\left\langle 1\right|]{\hat{D}}_{a}^{†}(\mu )=\frac{1}{2}\rho_{a}.$$

The trace of equation (4.5) is then


(4.6) $$\text{Tr}[\rho_{a}^{2}]=\frac{1}{2}\text{Tr}[\rho_{a}]=\frac{1}{4}\left({,}_{a}\langle 0|0\rangle_{a}+{,}_{a}\langle 1|1\rangle_{a}\right)=\frac{1}{2},$$

where we have utilized the cyclic property of the trace to resolve unity ${\hat{D}}_{a}^{†}(\mu ){\hat{D}}_{a}(\mu )\equiv 1$. This shows that the entanglement is independent of the parameters in the displacement operators, as expected given that the displacement operator action on a single field mode is a local operation that cannot increase entanglement. This is obviously the value we would obtain from the state on the right-hand side of equation (4.1). It is straightforward to generalize for the input state ${|N,\alpha \rangle }_{a,b}$, N>1, i.e. for arbitrary photon-number occupation in the a-mode.

## 5. Conclusions

The simple optical beam splitter can be used to demonstrate a wide variety of non-classical effects including entanglement [26], quantum interference in joint photon-number distributions [13] and much more. In this paper, we have demonstrated some of these effects and in particular showed how a single photon mixed with a coherent field can transform the statistical properties of the output of a beam splitter. We have also demonstrated that the entanglement of the output is independent of the amplitude of the coherent field.

In our work, we have been inspired by the work of Rodney Loudon, who left a lasting legacy of wonderful insights in the study of non-classical light that continue to this day. Our article is dedicated to his memory.

## Data Availability

This article has no additional data.
